# Nanocomposites of Natural Rubber Containing Montmorillonite Modified by Poly(2-oxazolines)

**DOI:** 10.3390/ma17164017

**Published:** 2024-08-13

**Authors:** Peter Boháč, Zuzana Nógellová, Miroslav Šlouf, Juraj Kronek, Ľuboš Jankovič, Hamed Peidayesh, Jana Madejová, Ivan Chodák

**Affiliations:** 1 Institute of Inorganic Chemistry, Slovak Academy of Sciences, Dúbravská cesta 9, 845 36 Bratislava, Slovakia; peter.bohac@savba.sk (P.B.); uachljan@savba.sk (Ľ.J.); uachjmad@savba.sk (J.M.); 2 Polymer Institute, Slovak Academy of Sciences, Dúbravská cesta 9, 845 41 Bratislava, Slovakia; zuzana.nogellova@savba.sk (Z.N.); juraj.kronek@savba.sk (J.K.); hamed.peidayesh@savba.sk (H.P.); 3 Institute of Macromolecular Chemistry, Czech Academy of Sciences, Heyrovského nám. 2, 162 06 Prague, Czech Republic; slouf@imc.cas.cz

**Keywords:** nanocomposites, organomodified montmorillonite, natural rubber, polyoxazolines

## Abstract

Nanocomposites with a natural rubber (NR) matrix containing organomodified montmorillonite (MMT) as a precursor of nanoparticles were prepared using two different polyoxazolines as surface modifiers of the MMT. The materials were characterized by X-ray diffraction, transmission electronic microscopy and ultimate mechanical properties, and parameters obtained by DMTA method (storage and loss moduli and loss tangent) were determined. It was found that the effect of nanofillers presence has a significant effect on tensile strength as well as elongation at break, which are higher for materials with higher viscosity due to the presence of carbon blacks compared to the composites without carbon blacks. From the two modifiers, poly(2-ethyl-2-oxazoline) was identified as a prospective modifier for surface modification of MMT used as the possible additive for tyre treads exhibiting optimal balance between fuel consumption and safety of driving concerning breaking action and lateral breakaway.

## 1. Introduction

Nanomaterials and nanotechnologies have attracted the interest of scientists for more than 30 years, and a number of important results have been published in this area [1]. Concerning the nanocomposites with polymeric matrices, nanoparticles and nanofibres have been investigated as suitable nanofillers in almost all kinds of plastics with very different outcomes [2].

Concerning polymer-based nanocomposites, two-dimensional nanoparticles, such as montmorillonite and halloysite represent the main interest as reinforcing nanofillers [3]. While for polyamides, the 2D nanoparticle addition appeared to be rather successful, polyolefins, especially polypropylene, are still not applied to a substantial extent. On the other hand, modification of the physical properties of thermosets such as epoxy or polyester resins proved to be rather successful in several potential applications.

The investigation of nanocomposites began with thermosets such as polyamides and polyolefins. The first successful data resulted in the extension of nanocomposite investigation to nanocomposites with elastomeric matrices, and the interesting outcomes have been published and, in many cases, applied also concerning natural rubber [4,5,6] as well as synthetic elastomers [7,8,9]. Considering the addition of organomodified MMT of nanoparticles, in many cases, the direct homogenization, including intercalation and exfoliation of clays to plate particulate fillers with extremely high surface area, is easier in rubbers compared to melt mixing of thermoplastics. There are two apparent reasons for this behaviour. First, standard preparation of rubber mixtures takes more time of processing in mixers or two-roll mills due to the necessity of homogeneously mixing the number of components, such as a vulcanizing system including sulphur, plasticizers, reinforcing fillers and other often special additives, making the whole process take more time than what is beneficial to a more homogeneous distribution of reinforcing particles. What seems even more effective in terms of the distribution of nanoparticles and the increase in their surface area seems to be the higher viscosity of rubbers compared to thermoplastics in a molten state so that the shear stress applied by the mixing device is transferred to particles more efficiently in rubbers. Therefore, it can be expected that the portion of intercalated and, especially, exfoliated particles might be higher after mixing the precursors of nanoparticles in a rubber matrix compared to molten thermoplastics or even liquid components of thermosets [10,11].

The reinforcement of natural rubber is quite frequently discussed, and several suggestions appear in the scientific literature, such as the substitution of carbon blacks [12,13] or an interesting method of emulsion stabilization destabilization [14]. If using MMT as the nanofiller, in all cases, a surface modification by organic modifier is needed to increase the distance between the individual layers in the nanofiller precursor [5,15]. The reason for this is to enable the intercalation of polymer chains inside the MMT structure and to support the exfoliation of the precursor to thinner particles with substantially higher surface area, as well as to decrease the exfoliated particles’ surface polarity resulting in a hindering the agglomeration and aggregation of the dispersed nanoparticles [5,15].

Various organomodifiers represent options for selecting optimal modification for each particular polymer—MMT pair [16]. Among the most interesting examples of optimization, a comparison of three modifiers may be shown. If MMT is modified by octadecyltrimethylammonium chloride (ODTA), octadecylammonium chloride (ODA) and oleylammonium chloride (OA), the highest increase in tensile strength and modulus in polyethylene occurred after MMT modified with ODTA as the largest cation, leading to the longest distance between the MMT layers, while the effects of ODA and OA were similar due to almost the same size of the intercalated cations. On the other hand, in natural rubber, the biggest effect was observed for OA, which was even higher compared to ODMA. In this case, during the vulcanization of rubber with sulphur, the benefit of a bigger size of cation was exceeded by the reaction of sulphur with a double bond present in OA, which results in a formation of covalent bonds of rubber chains with MMT surface modifier. This reaction does not proceed in natural rubber with an MMT surface modified by ODMA or ODA containing only fully saturated aliphatic moieties and no double bond available for direct addition to growing a rubber crosslinked network through reaction with sulphur. Thus, the size effect ruling the distance between the MMT layers is the only important factor concerning the ultimate properties of the nanocomposites [17].

It is seen that the application of various modifiers may bring unexpected effects concerning the ultimate properties of the polymer-based nanocomposite, resulting also in the number of useful applications [11]. From this point of view, MMT modification with species containing certain functional groups and possessing higher molar mass may affect the final properties in an unexpected way. If the cationic modifier contains functional groups able to substitute an atom on the surface of the MMT layer, the bulky cation may initiate the formation of greater distance between the parallel layers, making the following process of intercalation and/or exfoliation easier and more frequent. From this point of view, in this publication, we decided to test organic species with higher molecular weight, able to form cations that may substitute inorganic cations, especially Na^+^, on the surface of the MMT layers. Therefore, in this work, we synthesized poly(2-ethyl-2-oxazoline) and its partially hydrolysed analogue, which were used as surface modifiers of natrified MMT. Such modified clay was applied as a reinforcing filler to natural rubber, either without any other filler or also containing carbon blacks. The prepared mixtures were vulcanized, and the number of physical properties were tested. According to the results on properties of elastomeric matrix with a certain amount of organomodified MMT as a precursor of nanoparticles, the potential of MMT modified by the synthesized poly(2-oxazolines) might be estimated and considered for targeted application after optimizing the ultimate properties by adding the other components and selecting the appropriate method of preparation. In such a way, rubber mixtures were also applied for particular parts of tyres that could be designed for higher safety and lower fuel consumption.

## 2. Materials and Methods

### 2.1. Materials

The 2-ethyl-2-oxazoline (TCI Chemicals, Zwijndrecht, Belgium) was dried over potassium hydroxide for 48 h and distilled over calcium hydride under reduced pressure. Acetonitrile (Sigma-Aldrich, Steinheim, Germany) was dried and distilled over calcium hydride. Both chemicals were stored over molecular sieves 4Å (Merck, Darmstadt, Germany) under argon.

Natural rubber SMR CV 60 was supplied by Vegum, Dolne Vestenice, Slovakia. Highly purified sodium MMT (>98%) Kunipia-F (Na-KU), a product of Kunimine Industries Co., Ltd., Tokyo, Japan, of relatively high cation exchange capacity (CEC = 119 meq/100 g), was used as clay mineral. The sulphur-vulcanizing system consisted of stearic acid (Setuza, Ústí nad Labem, Czech Republic) and zinc oxide (Slovlak, Košeca, Slovakia) as activators, N-cyclohexyl-2-benzothiazole sulfenamide CBS (Duslo, Šaľa, Slovakia) as the accelerator, and sulphur (Siarkopol, Tarnobrzeg, Poland) as curing agent.

**Figure 1:**
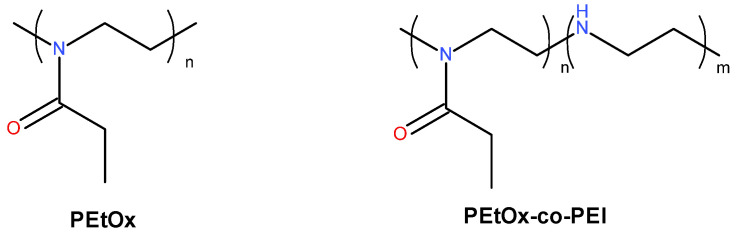
Chemical structures of poly(2-ethyl-2-oxazoline) (PEtOx) and statistical copolymer poly(2-ethyl-2-oxazoline-co-ethyleneimine) (PETOX-PEI).

Polymeric modifiers poly(2-ethyl-2-oxazoline) (PETOX) and statistical copolymer poly(2-ethyl-2-oxazoline-co-ethyleneimine) (PETOX-PEI) were synthesized as described in the next paragraph. The chemical formulas are shown in Figure 1. The molecular weight of the PETOX was approximately 14,800 g/mol, with dispersity Ɖ = 1.25, the molar ratio of copolymer components PETOX and polyethylene imine (PEI) was about 18:82, as determined from ^1^H NMR spectrum.

### 2.2. Synthesis of Poly(2-oxazolines)

**Figure 2:**
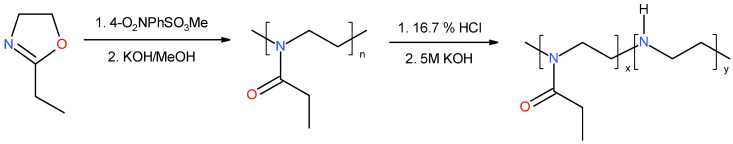
Scheme of synthesis of poly(2-ethyl-2-oxazoline) (PETOX) and statistical copolymer poly(2-ethyl-2-oxazoline-co-ethyleneimine) (PETOX-PEI).

The scheme of the procedure of synthesis of both modifiers is shown in Figure 2. In the first step, poly(2-ethyl-2-oxazoline) (PEtOx) was prepared by the cationic ring-opening polymerization of 2-ethyl-2-oxazoline initiated by methyl 4-nitrobenzenesulfonate (supplied by Sigma-Aldrich, Hamburg, Germany and used as received) according to a standard method [18]. 40 mL (0.4 mol) of 2-ethyl-2-oxazoline and 0.88 g (0.004 mol) of methyl 4-nitrobenzenesulfonate were dissolved in 40 mL of dry acetonitrile (c = 4 mol·dm^−3^) under argon atmosphere to achieve the theoretical degree of polymerization equal to 100. The polymerization was performed at 80 °C for 48 h, followed by the termination with 6 mL methanolic KOH (c = 0.1 mol·dm^–3^) in 1.5-fold excess at ambient temperature for 2 h. Resulting polymer was purified by the dialysis toward deionized water (Spectra/Pro 6, MWCO 1000 Da, Spectrum Laboratories, Rancho Dominguez, CA, USA). The structure of the resulting polymer was confirmed by ^1^H NMR spectrum (Figure S1).

The prepared PETOX was used as the core material for synthesis of the poly(2-ethyl-2-oxazoline-co-ethylene imine) (PETOX-PEI), which was prepared by partial hydrolysis of PETOX in 16.7% hydrochloric acid (purchased from Centralchem, Bratislava, Slovakia), as described elsewhere [19,20]. A total of 12.5 g of PETOX was dissolved in 250 mL 16 wt% aqueous HCl, and solution was heated at 110 °C for 2 h. Then, the reaction mixture was cooled in liquid nitrogen and neutralized with 5 M aqueous KOH. Partially hydrolysed polymer was purified by dialysis toward deionized water using dialysis membrane SpectraPro Nr.6 (MWCO 1 kDa, Spectrum Laboratories, USA) and then freeze-dried. The degree of hydrolysis of the copolymers was determined from ^1^H NMR spectrum (Figure S1).

For estimation of molar mass and dispersity, a SEC system consisting of Shimadzu LC-20, a Shimadzu refractive index detector and two PPS PFG 5 µm columns or PSS GRAM 5 µm columns from Polymer Standard Service GmbH (300 mm × 8 mm) at 25 °C was employed. DMAc with an addition of 0.1 wt% LiBr was used as an eluent at a flow rate of 1 mL·min^–1^, and samples were injected with a concentration of 1 mg·mL^–1^ Poly(methyl methacrylate) standards (Polymer Standard Services GmbH) were used for calibration.

^1^H NMR spectra were measured at room temperature on a Varian VXR-400 instrument (Varian, Wilmington, DE, USA) in CDCl_3_ solution using tetramethylsilane as an internal standard.

### 2.3. Modification of MMT by Poly(2-oxazolines)

The intercalation of either copolymer PEtOx-PEI or oligomer PEtOx into the Na-montmorillonite Kunipia was carried out by dispersing 10 g of montmorillonite particles (MMT) in 2000 mL of deionized water. The mixture was stirred overnight at room temperature using a magnetic stirrer. Subsequently, approximately the same molar amounts of the two oxazolines were dissolved in 200 mL of deionized water. The weight corresponding to a particular molar amount was calculated according to the molar mass of the monomeric unit, which was different for the two macromolecules. The prepared solutions were slowly added (5 mL/min) to the dispersion of Mt under extensive magnetic stirring (800 rpm) and maintained for seven days at room temperature. Mixed dispersions were transferred to the dialysis tubes and stored in deionized water for seven days. After dialysis, the dispersions were quickly frozen and lyophilized in a high vacuum. Two different concentrations of the modifiers were used to treat the virgin MMT, namely 100 or 300 miliequivalents of polymer per 100 g of clay, marked by the indicators 100-Mt or 300-Mt after the corresponding abbreviation of the particular polymer.

### 2.4. Preparation of Nanocomposites with MMT Modified by Poly(2-oxazolines)

The composition of the rubber mixture is shown in Table 1. Two different basic mixtures were prepared, both of the same composition except for one that contained, besides natural virgin rubber, solely all components of the vulcanizing system, while in the second 20 phr of carbon blacks, N-550 was added. The mixture was prepared in a 50 mL chamber of Brabender 331 at 80 °C, rotor speed of 30 rounds per minute. The time of mixing was 9.5 min for the mixture without CB, while in the second mixture after the mentioned time, 20 phr of carbon blacks were added, and mixing continued for additional 2 min. The sample undergoing the mixing process was further calendared to increase the homogeneity using a laboratory two-roll mill (Nishimura, Tokyo, Japan). The calendering process was conducted through sheeting, rolling, and sheeting steps at room temperature. This procedure was repeated four times with a rolling space of 5 mm, four times with a rolling space of 2 mm, and finally two times sheeting with a rolling space of 0.5 mm. After homogenization, vulcanization was performed in a laboratory press (Fontijne, Delft, The Netherlands) at 150 °C for 8 min.

### 2.5. X-ray Diffraction

X-ray diffraction (XRD) patterns were recorded in reflection mode, using a fixed sample stage for flat samples, on the EMPYREAN system (PANalytical B.V., Westborough, MA, USA), equipped with CuKα (λα1 = 1.54060 Å) radiation and operating at 45 kV and 40 mA. The patterns were scanned in the 2θ range 2.5–10° with scanning steps of 0.026° 2θ and scan step times of 100 s.

### 2.6. Transmission Electron Microscopy

Transmission electron microscopy (TEM) was employed in morphology visualization. All experiments were performed with a conventional TEM microscope (Tecnai G2 Spirit Twin; FEI, Prague, Czech Republic). The ultrathin sections for TEM microscopy were prepared by ultramicrotomy (ultramicrotome EM UC7; Leica, Vienna, Austria) at cryogenic conditions (sample and knife temperature were −80 and −50 °C, respectively). The ultrathin sections were collected on a standard TEM grid and observed by means of bright field imaging at the accelerating voltage of 120 kV.

### 2.7. Dynamic Mechanical Thermal Analysis (DMTA)

DMTA measurements of the nanocomposites were measured using a DMA Q800 (TA Instruments, Hüllhorst, Germany). The samples, ca. 10 × 7 × 1 mm^3^, were measured at a frequency of 10 Hz and an amplitude of 20 µm in tensile mode with a heating rate of 2 °C/min.

### 2.8. Mechanical Properties

The tensile properties of vulcanizates were measured using an Instron 3365 universal testing machine (Instron, Norwood, MA, USA) at cross-head speed of 50 mm/minute in uniaxial deformation at room temperature. The dumbbell-shaped test specimens (initial area of the deformed is part 3.5 × 30 mm, with thickness of approximately 1 mm) were used. The mean values and standard deviations were calculated from seven specimens for all parameters.

## 3. Results and Discussion

### 3.1. X-ray Diffraction

XRD is the standard method for evaluating the degree of intercalation (penetration of matrix polymer chains in between the individual clay layers). The lower the 2θ angle, the bigger the distance between the two layers and, consequently, the easier the chains of the matrix polymer penetrate inside the particle structure. Penetration of polymer chains inside the particles results in a stronger connection between the filler and the matrix, usually resulting in an increase in tensile strength. The increase in the distance between the layers also results in weakening the compactness of the particles so that a smaller shear load is needed to break the ordered structure of the layers, making exfoliation easier forming individual nanoparticles with substantially higher surface area. When all layers exfoliate, the ordered structure decays and all particles are exfoliated. If so, the peak in the region between 2θ angles 2 and 7 disappears.

**Figure 3:**
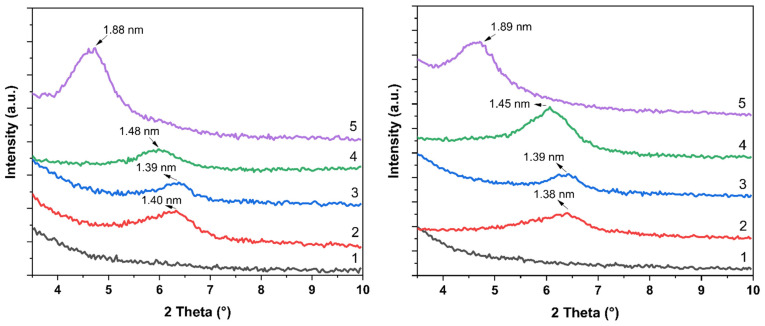
The XRD records of the NR vulcanizates without carbon blacks (left-hand side) and with 20 phr of N-550 carbon blacks (right-hand side) filled with 3 wt % of MMT modified with various amounts of modifiers, 1—no MMT, 2—100 meq of PEI-PETOX copolymer, 3—300 meq of PEI-PETOX copolymer, 4—100 meq of PETOX, 5—300 meq of PETOX.

The XRD records of our samples are shown in Figure 3. It is seen that if no MMT is present, no maximum appears in the 2θ angles region typical for the formation of ordered structures in MMT. The records of the samples with a content of 3 wt % of modified MMT exhibit the intercalation of modifier inside the space between the layers characterized by 2θ angles slightly over 6, corresponding to the distance of 1.38 to 1.40 nm. This is typical also for MMT without any organomodification, so the conclusion has been made that the copolymer PEI-PEtOx does not intercalate the MMT regardless of its smaller or higher content. On the other hand, in the presence of carbon blacks in the composite, certain exfoliation can be expected for lower content of PEI-PEtOx (100 meq) as indicated by the significantly broader shape of line 2 in the part from maximum to smaller 2θ angles, compared to the curve shape of the same MMT but containing higher content of the copolymer (line 3). A possible explanation of this effect may consist of the plasticization effect of PEI-PEtOx due to higher molecular weight and much lower content of carbonyl moieties compared to the PETOX modifier. The same conclusion is also made for the vulcanizate with carbon black content.

However, the vulcanizates filled with MMT modified with PEtOx behave differently. The effect of MMT modified with 100 meq of the oligomer PEtOx results in only a small, although significant, increase in the distance between the layers, but 300 meq of the PEtOx leads to an increase of about 60%.

Generally, the presence of carbon blacks does not substantially affect the behaviour of MMT with each particular organomodification. Modification by the copolymer PEI-PETOX does not exhibit any changes in interlayer distance, similar to the NR without carbon blacks. A smaller amount of PETOX brings about a certain increase in the distance, although this is smaller compared to the one in a mixture of NR without carbon blacks. It is of interest that the XRD data confirm that the distance in the NR nanocomposite does not depend on the presence or absence of carbon blacks in combination with MMT organomodified by two kinds of oxazolines as well as their equimolar content in the mixture.

### 3.2. TEM Microscopy

**Figure 4:**
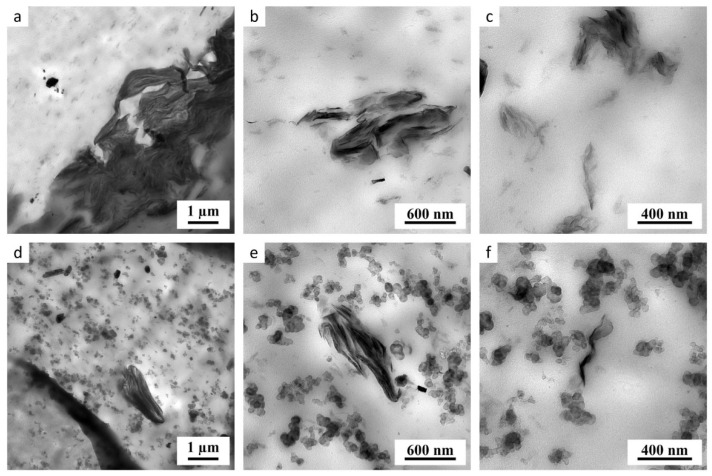
Examples of TEM micrographs showing typical morphologies in NR vulcanizates for samples of NR with 3 phr of MMT modified by PETOX100 both without (**a**–**c**) and with (**d**–**e**) carbon black. All samples, regardless of their modification, contained MMT in the form of large particles (**a**,**d**), smaller agglomerates (**b**,**e**), and exfoliated platelets (**c**,**f**). In contrast, the carbon black particles were homogeneously dispersed in all investigated samples (**d**–**f**).

The morphology of the prepared composites, i.e., the dispersion of the carbon black and MMT particles in the polymer matrix, was studied by TEM microscopy. The results are summarized in Figure 4, where the upper row (Figure 4a–c) shows NR vulcanizates without carbon black and the lower row (Figure 4d–f) shows NR with carbon black particles. In agreement with XRD results, the morphological differences among the samples with different types of modification (i.e., among the samples modified by 100 or 300 meq of PETOX-PEI or PETOX copolymer) were small. Even if the XRD results indicated that the modification by 300 meq of PETOX resulted in a 60% increase in the distance between the MMT layers, this effect could not be documented reliably in the TEM micrographs, as there were certain local variations in morphology. In other words, there were higher differences in morphology from place to place within each sample than among the individual samples. This is exemplified in Figure 4, where all investigated NR vulcanizates, both without and with carbon black, contained large MMT microparticles (Figure 4a,d), smaller MMT agglomerates (Figure 4b,e), as well as quite well-dispersed MMT platelets (Figure 4c,f). The dispersion of carbon black particles was very homogeneous in all samples (as also documented in Figure 4d–f).

### 3.3. Mechanical Properties

The data on mechanical properties belong to the basic information needed for prospective applications of any materials. In this work, tensile strength at break, elongation at break and three moduli, represented by values of tensile strength at 100, 200 and 300% of deformation, are shown in Table 2.

It is seen that the data for tensile strength at break and elongation at break more or less correspond, showing that the higher the elongation, the higher the tensile strength. This is in accordance with the mechanism of deformation behaviour of NR for which, at higher deformations, so-called elongation strengthening is observed, consisting of the extension of rubber chains, their orientation in the direction of load stress and a certain kind of crystallization.

The effect is also indirectly supported by the data of all moduli, which are almost the same for all modifications of MMT, indicating that the shape of the stress–strain curves is very similar not only regardless of the type of modification of MMT but also if comparing the NR containing MMT with the reference vulcanizate without MMT. At the beginning of the deformation, the load at 100% deformation is around 3 MPa; at 200%, it is around 6 MPa and at 300%, the load is somewhat above 9 MPa for all composites, including the reference sample. Thus, we may propose that the deformation of the investigated NR vulcanizates proceeds by a very similar mechanism; deformation is related to the load applied to the testing specimen, and the break is initiated by the presence of defects in the vulcanizate structure. In the case of composites with MMT content, the defect may be formed due to the presence of a few large aggregates of MMT. This may also explain the high scatter of values for both tensile strength as well as elongation at break for samples exhibiting high values of mechanical parameters. From this point of view, it is understandable that the scatter for samples with carbon black content is significantly lower since the carbon black content leads to higher viscosity of the material, resulting in higher shear during mixing so that the probability of large aggregate formation is much lower.

### 3.4. Dynamic Mechanical Thermal Analysis (DMTA)

Dynamic mechanical, thermal analysis is used mainly for the determination of transition temperatures of materials. Concerning plastics, most frequently, the glass transition temperature is determined as the maximum on the temperature dependence of loss tangent tan δ, being the ratio of loss modulus G″ and storage modulus G′ (tan δ = G″/G′). Besides this, the number of other data can be determined and identified from the temperature dependence of loss tangent on temperature.

**Figure 5:**
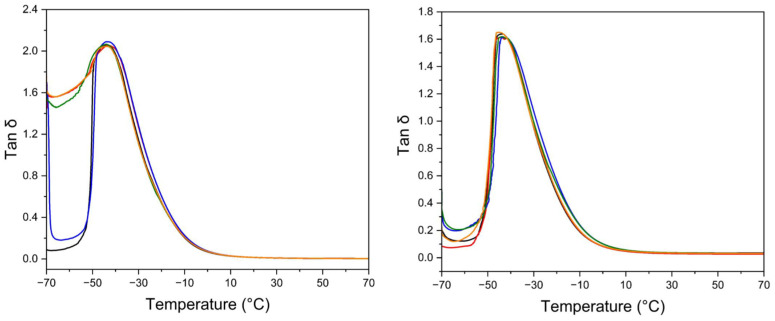
The DMTA records of the NR vulcanizates without carbon blacks (left-hand side) and with 20 phr of N-550 carbon blacks (right-hand side) filled with 3 wt% of MMT modified with various amounts of modifiers, black—no MMT, red—100 meq of PEI-PETOX copolymer, blue—300 meq of PEI-PETOX copolymer, green—100 meq of PETOX, orange—300 meq of PETOX.

In our case, the dependences of tan δ on temperature are almost identical for all materials investigated, as seen in Figure 5. Corresponding glass transition temperatures are very close to each other, as seen in the last column in Table 2. This means that the presence of MMT in any kind of organomodification does not have any effect on the mobility of the rubber chains, which are not directly connected with the MMT surface. The MMT either does not create strong bonds with the rubber segments, or the bonds, of whatever strength, are located close together and interact only with a limited number of rubber segments. In the latter case, interactions with MMT present themselves similar to chemical crosslinks by sulphur, resulting in reinforcement of the structure and an increase in tensile strength. In the case of weak interactions, the main effect consists of the formation of defects leading to premature fracture during unidirectional tensile test.

However, DMTA is also a very useful tool to evaluate the quality of rubber vulcanizates used for the tread of tyres. The method consists of measuring the loss tangent value at various temperatures, and the data indicate the loss of energy during driving. Taking this into account and realizing that the temperature of the tyre during driving is between 60 to 80 °C and the frequency of the cyclic deformation 10 Hz corresponds to the speed of the vehicle around 100 km/h, the loss tangent is related to the part of fuel consumption spent for overcoming the rolling resistance. On the other hand, while this parameter should be as low as possible to save fuel, the tan δ at temperature 0 to 20 °C indicates the energy loss during braking action and eliminating lateral breakaway of the vehicle, so the values of energy loss should be as high as possible. Therefore, for small fuel consumption but safe driving in curves and on icy surfaces, the tan δ value should be low between 60 to 80 °C and high between 0 and 20 °C. Additives that help adjust the ultimate properties, as indicated above, are permanently sought by tyre-producing companies.

The data in Table 3 summarize the behaviour of natural rubber with the addition of MMT modified in four different ways. It is seen that the values of tan δ for vulcanizate without carbon blacks are identical at 20 °C while the values at 0 °C are slightly higher; for the vulcanizate with 20 phr of carbon blacks, the values for PEI-PETOX 300 and PETOX 100 are more than 20% higher compared to the reference sample. At the same time, these samples exhibit only a marginal decrease in tan δ at 60 and 80 °C. Considering the fact that the tensile strength of PETOX 100 is the highest of all investigated samples, this material might be selected as the most promising for the broad range of applications.

## 4. Conclusions

The addition of precursors of the formation of two-dimensional nanoparticles modified by oligomeric poly(2-oxazolines) modifiers results in interesting properties with possible application benefits.

1. The mixture containing carbon blacks exhibits higher absolute as well as relative increases in tensile strength compared to the mixture composition without carbon blacks. The reason for this is seen in the higher viscosity of the rubber mixture containing carbon blacks, resulting in higher shear applied to nanoparticles during the mixing of the elastomer blend, leading to a higher portion of intercalated and/or exfoliated particles in the final material.
2. The oligomeric modifiers of the MMT surface represent a useful contribution to certain parameters if NR composition is optimized for a particular application. Tyre tread was presented as an example of such an effect.
3. Although almost no changes in glass transition temperature were observed, the values of loss tangent indicate that if an optimal composition of mixture for tyre tread would be formed, the presence of MMT organomodified with oligomeric poly(2-oxazolines) being a part of the mixture may be beneficial for balance between fuel consumption and driving safety.
4. Our data indicate that the best performance was detected for MMT modified by the lower amount of polyoxazoline PETOX considering the DMTA results as well as the highest tensile strength in mixtures with carbon blacks (being the same as the mixture containing the MMT modified with PEI-PETOX copolymer).

## Figures and Tables

**Table 1:** Composition of the rubber mixture. NR—natural rubber, CB—carbon blacks, MMT—montmorillonite, phr—per hundred parts of the rubber, CBS—N-cyclohexyl-2-benzothiazole sulfenamide.

Component	phr
NR	100
CB N550	0 or 20
filler MMT	0 or 3
Sulfenax CBS	3.5
ZnO	5
Stearic acid	2
Sulphur	3.5

**Table 2:** Tensile strength at break (σ), elongation at break (ε) and moduli M100, M200 and M300, as well as glass transition temperature T_g_ for vulcanizates containing 3 phr of MMT with various modifications and their different content. Upper part—vulcanizates without carbon blacks, lower part, vulcanizates with 20 phr of carbon blacks N550.

	MMT 3 phr	Tensile Strength σ (MPa)	Elongation at Break ε (%)	M100 (MPa)	M200 (MPa)	M300 (MPa)	T_g_ (°C)
Without CB	no	6.9 ± 2.6	397 ± 87	1.62 ± 0.02	2.84 ± 0.05	4.46 ± 0.08	−43.2
	PEI-PETOX 100	8.8 ± 4.5	433 ± 150	1.73 ± 0.03	2.96 ± 0.04	4.60 ± 0.06	−43.2
	PEI-PETOX 300	2.3 ± 0.3	155 ± 25	1.66 ± 0.04	n/a	n/a	−44.3
	PETOX 100	2.6 ± 0.3	181 ± 26	1.63 ± 0.02	n/a	n/a	−44.1
	PETOX 300	6.2 ± 2.8	369 ± 124	1.68 ±0.03	2.86 ± 0.06	4.39 ± 0.07	−44.0
Filled with 20 phr CB	no	13.3 ± 0.9	405 ± 30	2.89 ± 0.02	5.77 ± 0.04	9.2 ± 0.1	−43.8
	PEI-PETOX 100	17.3 ± 1.4	461 ± 31	3.08 ± 0.09	6.07 ± 0.18	9.8 ± 0.3	−44.1
	PEI-PETOX 300	10.2 ± 1.2	328 ± 32	2.92 ± 0.04	5.70 ± 0.07	9.1 ± 0.1	−44.2
	PETOX 100	17.2 ± 1.7	457 ± 43	2.98 ± 0.05	5.97 ± 0.12	9.7 ± 0.2	−43.8
	PETOX 300	14.5 ± 2.0	400 ± 41	2.01 ± 0.02	5.08 ± 0.06	9.8 ± 0.2	−45.4

**Table 3:** Temperatures of maximum values for tan δ appearance as extracted from Figure 4. Upper part—vulcanizates without carbon blacks, lower part—vulcanizates with 20 phr of carbon blacks N550.

	MMT 3 phr	Tan Delta			Tan Delta at 78.2 °C
		at 0 °C	at 20 °C	at 60 °C	
Without CB	no	0.068	0.015	0.0060	0.0046
	PEI-PETOX 100	0.076	0.015	0.0059	0.0045
	PEI-PETOX 300	0.077	0.015	0.0043	0.0038
	PETOX 100	0.072	0.015	0.0045	0.0037
	PETOX 300	0.073	0.015	0.0052	0.0041
Filled with 20 phr CB	no	0.084	0.041	0.0350	0.035
	PEI-PETOX 100	0.083	0.035	0.0280	0.027
	PEI-PETOX 300	0.103	0.042	0.0320	0.032
	PETOX 100	0.102	0.042	0.0330	0.032
	PETOX 300	0.086	0.039	0.0320	0.031

## Data Availability

The original contributions presented in the study are included in the article, further inquiries can be directed to the corresponding author.

## Supplementary Materials

The following supporting information can be downloaded at: https://www.mdpi.com/article/10.3390/ma17164017/s1, Figure S1: Elugram of poly(2-ethyl-2-oxazoline) recorded by gel permeation chromatography.
