# Supplementary figures and images for: Nanocomposites of Natural Rubber Containing Montmorillonite Modified by Poly(2-oxazolines)
Source: Materials (Basel). 2024 Aug 13;17(16):4017. doi: 10.3390/ma17164017 (PMC11356297; doi:10.3390/ma17164017)

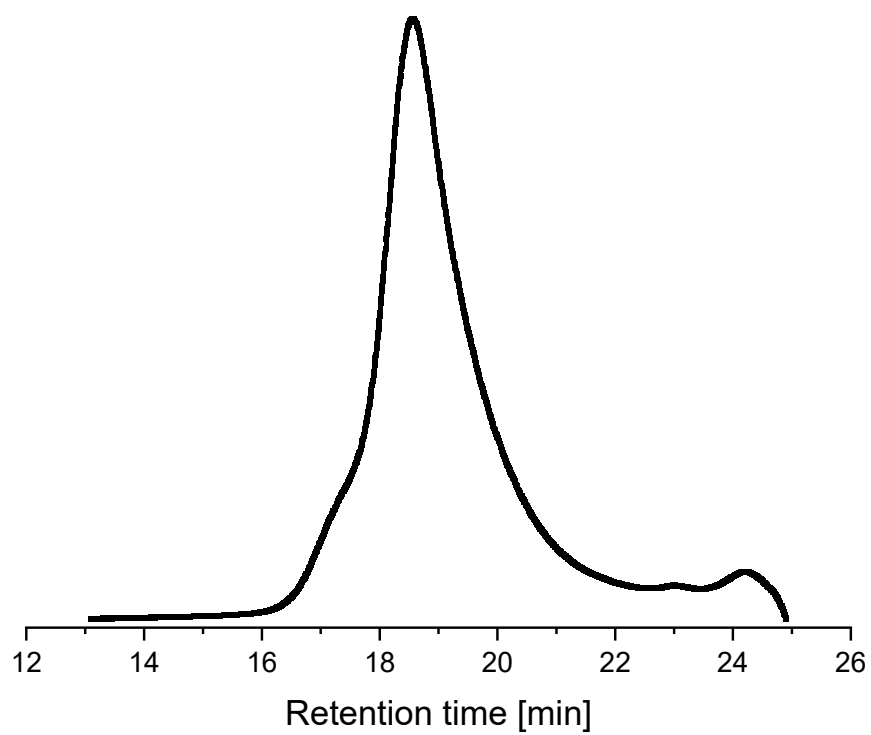

**Figure S1.** Elugram of poly(2-ethyl-2-oxazoline) recorded by gel permeation chromatography.

Supplement: Supplementary file 1 [file materials-17-04017-s001.zip › materials-3074355-supplementary.pdf]
